# Heart rate variability biofeedback for critical illness polyneuropathy: a randomized sham‐controlled study

**DOI:** 10.1111/ene.16512

**Published:** 2024-10-18

**Authors:** Annahita Sedghi, Christoph Bartels, Erik Simon, Florian Krause, Martin Arndt, Stefan Zsigri, Kristian Barlinn, Ulf Bodechtel, Ana Isabel Penzlin, Timo Siepmann

**Affiliations:** ^1^ Dresden Neurovascular Center, Department of Neurology, Medical Faculty and University Hospital Carl Gustav Carus TUD Dresden University of Technology Dresden Germany; ^2^ Department of Neurology and Rehabilitation Klinik Bavaria Kreischa Kreischa Germany; ^3^ Department of Internal Medicine 1, Medical Faculty and University Hospital Carl Gustav Carus TUD Dresden University of Technology Dresden Germany; ^4^ Department of Intensive Care Medicine and Weaning Klinik Bavaria Kreischa Kreischa Germany; ^5^ Department of Neurology Rhön Klinikum Campus Bad Neustadt Bad Neustadt Germany

**Keywords:** biofeedback, heart rate variability, neuropathy, parasympathetic, sepsis

## Abstract

**Background and purpose:**

Critical illness polyneuropathy (CIP) has been linked to neurocardiac dysfunction mediated by autonomic nervous system dysregulation, which increases mortality. We aimed to assess if heart rate variability (HRV) biofeedback could improve neurocardiac function in CIP.

**Methods:**

We randomly allocated (1:1) patients with electrophysiologically confirmed CIP undergoing early inpatient neurological rehabilitation to additional HRV or sham biofeedback over 14 days. We evaluated neurocardiac function via standard deviation of normal‐to‐normal intervals (SDNN) as the primary outcome, as well as HRV frequency domains, sympathetic cutaneous sudomotor and vasomotor functions and disability at baseline, post intervention and 4 weeks later. The study is registered on the German Clinical Trials Register (DRKS00028911).

**Results:**

We included 30 patients with CIP (40% females, median [interquartile range] age 64.6 [56, 72] years). We observed an increase in SDNN and the predominantly parasympathetic high frequency domain post intervention (*ß* = 16.4, 95% confidence interval [CI] 0.2, 32.6 [*p* = 0.047] and *ß* = 1179.2, 95% CI 119.9, 2158.5 [*p* = 0.018]), which was sustained at the 4‐week follow‐up (*ß* = 25.7, 95% CI 6.0, 45.4 [*p* = 0.011] and *ß* = 25.7, 95% CI 6.0, 45.4 [*p* = 0.011]). Patients who underwent HRV biofeedback displayed a higher adjusted Barthel index, indicating less severe disability 4 weeks after the intervention compared to those in the sham group (*ß* = 23.3, 95% CI 5.5, 41.1 [*p* = 0.014]). Low frequency and sympathetic skin functions did not differ between groups (*p* = nonsignificant).

**Conclusions:**

Our study provides pilot data suggesting that, in patients with CIP, HRV biofeedback can improve neurocardiac function with a predominant effect on the parasympathetic nervous system and has a beneficial effect on functional recovery.

## Graphical abstract

Our randomized controlled pilot study in patients with critical illness polyneuropathy after sepsis undergoing early in‐patient neurological rehabilitation provides pilot data suggesting that heart rate variability biofeedback can improve neurocardiac function with a predominant effect on the parasympathetic nervous system. We also observed a beneficial effect on regaining functional independence in this severely ill population.

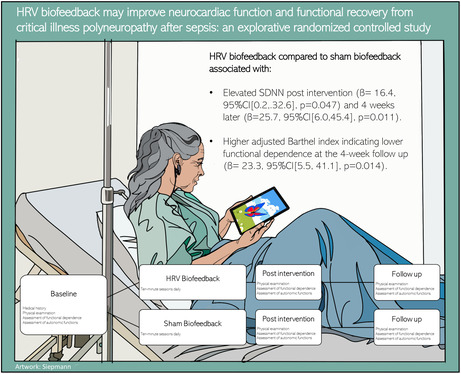

## INTRODUCTION

Critical illness polyneuropathy (CIP) is a common complication of severe illness, affecting up to half of patients undergoing critical care and up to two thirds of those with sepsis [1, 2]. Patients with CIP experience symmetric and flaccid limb weakness as well as weakness of the muscles used for breathing, which worsens clinical outcome and increases mortality [2, 3]. Over the past four decades, research has explored the pathophysiology of CIP and identified multiple mechanisms leading to axonal sensorimotor nerve damage in somatic nerves of critically ill patients including impaired neural excitability, peripheral axon death, impaired ionic balance, failure of neuromuscular transmission and exacerbated systemic inflammation [3]. Involvement of the autonomic nervous system in CIP is less well studied on a pathophysiological level. However, disturbances of neurocardiac dysfunction with reduced heart rate variability (HRV) are seen in up to 100% of critically ill patients, including those with weakness acquired during intensive care unit stay, independently predicting in‐hospital mortality in those who are affected by sepsis [4, 5]. Cumulative evidence demonstrated that reduced HRV reflecting dysfunction of the autonomic neurocardiac system worsens clinical outcomes in patients with sepsis as well as critical neurological and cardiovascular diseases [6]. The pathophysiological links, whereby low HRV is associated with poor neurological outcome in these disorders, are complex and encompass changes to cerebral perfusion, neurogenic regulation of cardiac myoctes, and increased hyperinflammatory and anti‐inflammatory responses [7].

Currently, neither acute nor long‐term rehabilitative treatment regimens of CIP comprise targeted therapy of neurocardiac dysfunction. This can be explained by a paucity of data on specific autonomic treatment in this population. Non‐invasive, non‐pharmacological modulation of neurocardiac function to increase HRV can be achieved through HRV biofeedback. This technique uses a metronomic breathing pattern to elevate vagal tone and increase HRV with continuous quantification and visualization of HRV on a digital screen in real time [8]. Previous randomized studies demonstrated that HRV biofeedback can improve neurocardiac function by enhancing vagal heart rate control to elevate HRV in patients with coronary artery disease and acute ischemic stroke [8, 9].

We aimed to test the hypothesis that HRV biofeedback can improve neurocardiac function by enhancing vagal control in patients with CIP after sepsis. Furthermore, we aimed to assemble pilot data on the potentially beneficial effect of HRV biofeedback on functional recovery in this severely ill population of patients.

## METHODS

### Study design

We performed a randomized, sham‐controlled study at the neurological early rehabilitation unit of a multidisciplinary 1250‐bed inpatient rehabilitation hospital with a comprehensive sepsis center in Germany.

### Study population

We included adult patients undergoing early inpatient neurological rehabilitation after sepsis, with a diagnosis of CIP confirmed on nerve conduction study. To minimize confounders of autonomic function evaluation we excluded patients with clinical evidence or history of autonomic or diabetic neuropathy, atrial fibrillation, chronic obstructive lung disease, addiction to alcohol, or intake of tricyclic antidepressants within the last 14 days. We excluded those who were unable to participate in HRV biofeedback training due to cognitive impairment, aphasia, insufficient respiratory function, blindness, deafness or other forms of functional impairment leading to the inability to participate in the intervention.

### Study protocol

We allocated patients with CIP following sepsis in a random sequence (1:1) to undergo either daily 10‐min sessions of HRV biofeedback or sham biofeedback over a period of 14 days, in addition to standard early inpatient neurological rehabilitation. The sequence of allocation was determined by an investigator (C.B.) using an online randomization platform (randomizer.org). We used numbered containers to conceal the sequence until interventions were assigned to our study participants. An investigator (C.B.) recruited patients between June 2021 and July 2023 and assigned them to interventions. We obtained medical history and conducted a physical examination, including assessment of neurological functions, and performed quantitative autonomic neurocardiac, cutaneous vasomotor and sudomotor testing in all patients at study entry (baseline). In patients allocated to the intervention study arm, HRV biofeedback was performed over 10 min daily for 14 consecutive days. Those in the control study arm underwent sham biofeedback for the same duration and frequency. Patients were blinded to group allocation (sham control or biofeedback). We repeated assessment of autonomic functions and symptoms immediately after the last day of biofeedback training (post intervention) as well as 4 weeks later (follow‐up). We assessed disability at all time points of evaluation.

**FIGURE 1:**
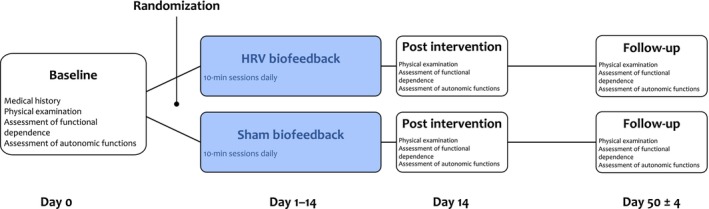
Study timeline. Timeline and sequence of assessments and study interventions. We evaluated neurovascular autonomic function immediately after the last heart rate variability (HRV) biofeedback or sham training on Day 14.

Study interventions were carried out by a board‐certified neurologist with expertise in rehabilitation medicine who was not blinded to treatment allocation (C.B.) because of the necessity to instruct the patient and monitor that they were performing the biofeedback training accurately. An investigator (A.S.) who was blinded to group allocation performed statistical analysis. The study timeline is shown in Figure 1.

### Standard inpatient rehabilitation care

Early neurological rehabilitation combined intensive medical care with multidisciplinary stimulating rehabilitative treatment including speech and swallowing therapy, physical therapy and occupational therapy. The aim of this standardized multimodal treatment is to recover basic functions including awareness, stabilization, ability to communicate, swallowing, mobility, cognitive functions, coordination and the ability to perform activities of daily living.

### Study intervention: HRV biofeedback and sham biofeedback

We performed HRV biofeedback as previously described. [10] In brief, we instructed patients to breath with a frequency of six breathing cycles/min to facilitate respiratory sinus arrhythmia and thereby elevate the amplitude of heart rate oscillations. The consequential increase in HRV was measured continuously by a biofeedback system (Stress Pilot Manager®; BITsoft Health Systems GmbH, Bitburg, Germany) using an ear pulse sensor. Dynamic changes of HRV during the breathing exercise were visualized on a computer screen as a hovering balloon. The balloon ascended with increasing HRV and descended with decreasing HRV to provide direct visual feedback in real time. Breathing instructions were given and visualized as a moving bar on the screen, with upward movement indicating the need to breath in and downward movement to breath out. Patients underwent 10‐min sessions of biofeedback training once daily over a period of 14 days. We initially intended to set the duration of each training session at 20 min but preliminary test runs showed that CIP patients may lack sufficient endurance in metronomic breathing. Therefore, we decided to perform 10‐min training sessions, which is consistent with previous studies on HRV biofeedback in patients with stroke and coronary heart disease [8, 9]. Before the first training session, patients underwent an introduction and test session to ensure proper execution of training and to improve adherence. An investigator (C.B.) attended and monitored each training session with all study patients to ensure compliance with the intervention protocol.

Patients allocated to the control group received sham biofeedback sessions of identical duration, frequency and setting. During sessions they also looked at the computer screen displaying a balloon, but they did not receive any breathing instructions and neither was their HRV measured or linked to the balloon's shown altitude.

### Evaluation of autonomic functions

We used a data acquisition system (Power‐Lab®; ADInstruments, Castle Hill, Australia) with amplifiers of biosignals for ECG signals (Single Bio Amp® FE231; ADInstruments) and sympathetic skin response (GSR Amp® FE116; ADInstruments). Neurovascular skin function was evaluated using a laser Doppler flowmeter with a fiber optic probe (Blood FlowMeter®; ADInstruments). We analyzed all biosignals using the software package LabChart Pro® version 8 for Windows (ADInstruments). All study participants underwent autonomic testing in the patient's room in our comprehensive inpatient rehabilitation center, with a room temperature of 20°C–23°C, in a semi‐recumbent or sitting position after a 10‐min lasting rest.

### Evaluation of neurocardiac function

We computed HRV to assess autonomic neurocardiac function from continuous recording of cardiac electrical activity over two phases of 3 min each using a three‐channel electrocardiogram. In Phase 1, we instructed patients to breathe normally, that is, at a spontaneous frequency under resting conditions. In Phase 2, patients were instructed to breath at a metronomic frequency of six breathing cycles per min. The inspiration/expiration ratio was set to 1.5/1 as this elevates parasympathetic tone to increase HRV. This breathing pattern was indicated to the patient via a moving bar on the screen, where upward movement indicated the requirement to breath in and downward movement to breath out. Analysis of HRV parameters were conducted for both phases of recording separately. We performed time domain analysis of HRV by computing the standard deviation of normal beat‐to‐beat intervals (SDNN). This parameter is primarily influenced by parasympathetically mediated respiratory sinus arrhythmia in short‐term recordings under resting conditions. We also computed root mean square of successive RR interval differences (RMSSD), a time domain parameter of HRV that is predominantly influenced by parasympathetic tone but less affected by respiration [11].

Furthermore, we performed power spectral analysis of HRV using fast Fourier transformation to assess frequency bands, as described elsewhere [12]. Spectral components comprised high frequency and low frequency.

### Evaluation of neurovascular autonomic function

We performed laser Doppler flowmetry to assess skin blood flow on the tip of the index finger of the non‐dominant hand after sympathetic stimulation, as described elsewhere [13]. In brief, infrared light at a wavelength of 950 nm was emitted by a diode. While transmitting through the finger, the light was partially absorbed. A sensor captured the remaining light continuously and produced an electric current, the intensity of which was in proportion to the energy of non‐absorbed light, reflecting dynamic changes in cutaneous blood volume. We evaluated vasoconstriction of skin blood vessels at 2‐mm depth in response to forced deep inspiration with temporal resolution. Vasoconstrictory response was defined as the ratio of the blood flow at baseline minus the lowest blood flow after deep inspiration and blood flow at baseline.

### Evaluation of sudomotor autonomic function

We assessed sympathetic skin response to study sudomotor autonomic skin function as previously described [14]. Briefly, we measured skin conductance over time with two finger electrodes on the fingertips III and IV of the non‐dominant hand. Sympathetic skin response was defined as maximum increase in skin conductance following forced deep inspiration.

### Functional outcome

We assessed activities using the Barthel index to evaluate disability, as described elsewhere [15]. This ordinal scale evaluates functional dependence in the domains of mobility and personal care in people with a disabling, chronic condition in a rehabilitation setting.

### Statistical analysis

All analyses were performed using the statistical software package Stata (version 17.0 MP‐Parallel Edition; College Station, TX, USA). Outcome variables were checked for normality using descriptive (skewness, kurtosis) and analytical (Shapiro–Wilk test) criteria. The significance level was set at *α* = 0.05. No sample size calculation or correction for multiple comparisons was performed due to the exploratory character of study. Missing data were not imputed. Available case analysis was performed. After descriptive checking for normality of continuous variables, we displayed mean or median with standard deviation (SD) or interquartile range (IQR), respectively, where appropriate. Between‐group differences at baseline were assessed with non‐parametric Mann–Whitney *U* tests for non‐normally distributed continuous or ordinal data. Fisher's exact test was used for count data. Analyses were performed using multilevel linear mixed models for each investigated outcome variable including HRV, sympathetic skin response and laser Doppler flowmetry parameters. Main effects of group, time, and time × group interaction were computed. Group and time were declared as fixed effects. Patient ID was declared as a random effect with a random intercept. Main effects were adjusted for covariates age, sex, Barthel's index score at baseline, beta‐blocker use, arterial hypertension, diabetes, obesity, chronic kidney failure, COVID‐19 comorbidity at disease onset, and approximate disease duration (time from hospitalization for acute illness). The effect of HRV biofeedback compared to sham intervention on disability at follow‐up was assessed using multivariable regression with adjustment for the same covariates, with the exception of beta‐blocker use. Residuals were tested for normality using the qnorm function. Where the assumption of normality was not fulfilled due to extreme values, robust standard errors were calculated. Extreme values were not defined as outliers due to an expected high inter‐personal variability of the parameters by nature. Margin and contrasts of margins were plotted to visualize interactions.

### Standard protocol approvals, registrations, and patient consents

Our study was approved by the local institutional review board (*Ethikkommission an der TU Dresden*, IRB reference number: EK 474102019). The study protocol was provided to the German Clinical Trials Registry (DRKS00028911). Written and oral informed consent for participation was obtained from each study participant prior to inclusion. The CONSORT checklist is shown in Data S1.

## RESULTS

### Study population

**FIGURE 2:**
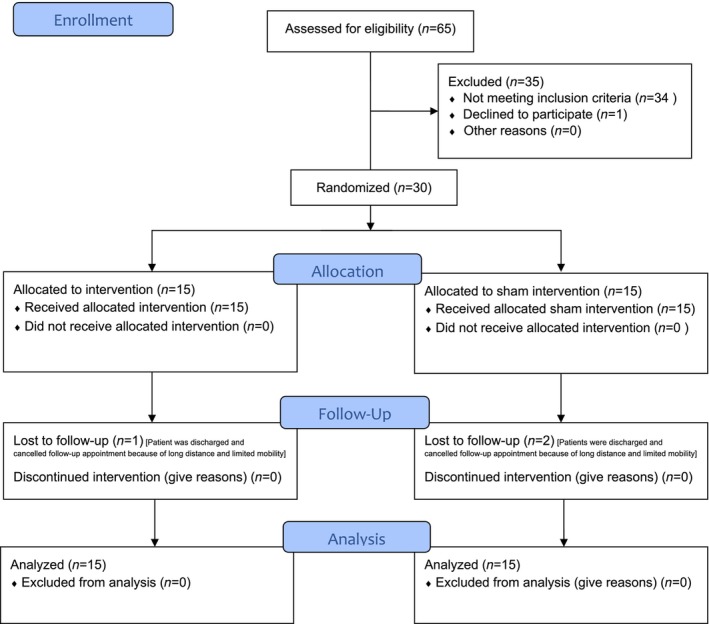
CONSORT 2020 flow diagram. Flowchart according to the updated 2010 CONSORT statement (accessed May 5, 2024. https://www.equator‐network.org/reporting‐guidelines/consort/).

We included 30 patients with CIP (40% females, median [IQR] age 64.6 [56, 72] years). Both groups (HRV biofeedback and sham biofeedback) were balanced for demographic and disease characteristics as well as for cardiovascular risk profiles and comorbidities, as shown in Table 1. We did not reach our pre‐specified recruitment goal of 48 patients because of institutional restrictions during the COVID‐19 pandemic. A flow diagram of the progress through the phases of our study is shown in Figure 2. The results of nerve conduction studies in each study participant are shown in Supplementary Information S1.

**TABLE 1:** Demographic and baseline characteristics.

	Sham biofeedback	HRV biofeedback	*p* value
Sex: female, *n* (%)	7 (46.7)	5 (33.3)	0.71
Age, median (IQR) years	69 (55, 77)	64 (56, 67)	0.57
Time between initial hospitalization for acute illness and hospitalization at the rehabilitation center, median (IQR) days	55 (24, 63)	44 (30, 89)	0.91
Duration spent in rehabilitation, median (IQR) days	108 (69, 138)	120 (61, 194)	0.29
Barthel index, median (IQR) on admission	15 (0, 45)	20 (5, 35)	0.96
Barthel index, median (IQR) on discharge	60 (45, 70)	65 (50, 75)	0.54
Polyneuropathy according to type of nerve damage, *n* (%)			1.00
Axonal and demyelinating	7 (46.7)	7 (46.67)	
Axonal	8 (53.3)	7 (46.67)	
Demyelinating	0 (0)	1 (6.67)	
Polyneuropathy according to somatic system, *n* (%)			1.00
Motor and sensory	14 (86.7)	14 (93.3)	
Motor	2 (12.3)	1 (6.7)	
Sensory	0 (0)	0 (0)	
Beta blocker, *n* (%)	9 (60)	8 (53.3)	1.00
Arterial hypertension, *n* (%)	9 (60.0)	7 (46.67)	0.72
Diabetes mellitus, *n* (%)	7 (46.67)	7 (46.67)	1.00
Chronic renal failure, *n* (%)	4 (26.7)	6 (40.0)	0.70
Smoking	0 (0)	0 (0)	1.00
Obesity, *n* (%)	5 (33.3)	7 (60.0)	0.27
COVID‐19 immediately prior to admission, *n* (%)	3 (20.0)	5 (33.3)	0.68

*Note*: Table of demographic and baseline characteristics including cardiovascular risk profiles. Due to the small sample size group comparisons were undertaken via Mann–Whitney *U* test for non‐normally distributed continuous / ordinal data count data and Fisher's exact test for count data.

Abbreviations: COVID‐19, coronavirus disease 2019; HRV, heart rate variability; IQR, interquartile range; *n*, number.

### Missing data, safety and adherence

Our dataset was complete at baseline and post intervention. Missing data at follow‐up were limited to three patients (10%), who were lost to follow‐up. Assessment of HRV under paced breathing at follow‐up was missing in two additional patients due to their failure to execute breathing instructions. Ten patients (five in the HRV biofeedback group and five in the sham group) reported difficulties performing paced breathing during assessment but none of our patients reported difficulties during HRV biofeedback. No adverse or serious adverse events were noted. All study patients completed the intervention or sham intervention.

### Neurocardiac function

**FIGURE 3:**
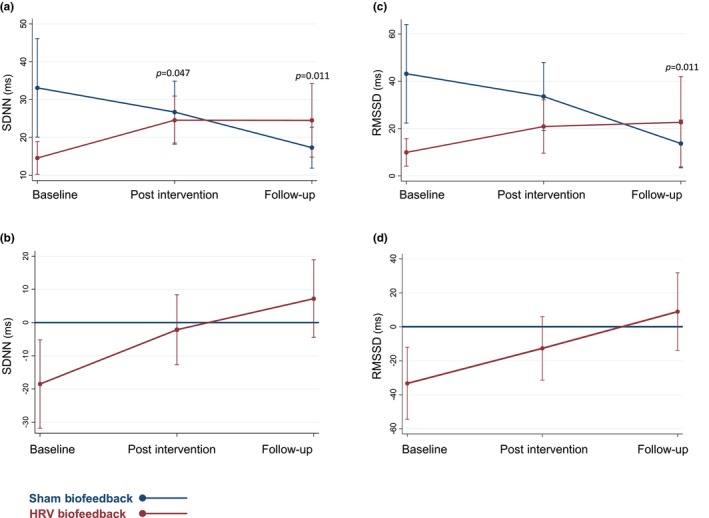
Time domain analysis of heart rate variability (HRV) under normal breathing. Time domain parameters of HRV under normal breathing. Predictive margins plots of SDNN (a), and root mean square of successive RR interval differences (RMSSD) (c) showing linear predictions with fixed proportions with 95% confidence intervals. Contrast of predictive margins plots for standard deviation of normal‐to‐normal intervals (SDNN) (b), and RMSSD (d) with 95% confidence intervals. ms, milliseconds.

We observed an increase in HRV assessed via the primary outcome SDNN under normal breathing after HRV biofeedback compared to the sham intervention, indicated by an interaction effect between treatment group and measurement time point at post‐intervention assessment (*ß* = 16.4, 95% confidence interval [CI] 0.2, 32.6; *p* = 0.047) as well as follow‐up (*ß* = 25.7, 95% CI 6.0, 45.4; *p* = 0.011). Patients in the sham biofeedback group displayed a decline in SDNN over time (Figure 3a,b). When assessing HRV via RMSSD under normal breathing conditions, we also observed an increase after HRV biofeedback compared to the sham group, reaching statistical significance at follow‐up (*ß* = 42.2, 95% CI −53, −5.2; *p* = 0.011 [Figure 3c,d]). Arterial hypertension emerged as an independent predictor for low RMSSD and SDNN values under normal breathing (*ß* = −16.5, 95% CI −33.0, −0.1 [*p* = 0.049] and *ß* = −9.7, 95% CI −17.5, −1.8 [*p* = 0.02], respectively). We did not observe any changes in time domain parameters of HRV after the study intervention or the sham intervention when assessed under paced breathing (Table S2). However, arterial hypertension remained an independent predictor for low RMSSD and SDNN values (*ß* = −23.1, 95% CI −42.1, −4.2 [*p* = 0.017] and *ß* = −13.0, 95% CI −26.0, 0.02 [*p* = 0.050], respectively) under paced breathing.

**FIGURE 4:**
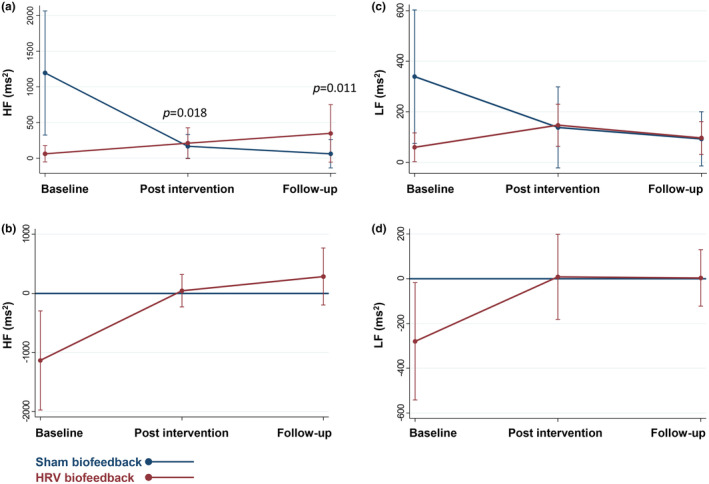
Frequency domain analysis of heart rate variability (HRV) under normal breathing. Predictive margin plots of high frequency (HF) band of HRV (a), a and low frequency (LF) band of HRV (c) showing linear predictions with fixed proportions with 95% confidence intervals. Contrasts of predictive margins plots for HF band of HRV (b), and LF band of HRV (d) HF with 95% confidence intervals. ms, milliseconds.

On spectral analysis of HRV, patients undergoing HRV biofeedback achieved an increase of high frequency compared to those who underwent sham training with a positive interaction effect between treatment group and measurement time point at the time of follow‐up (*ß* = 1179.2, 95% CI 119.9, 2158.5; *p* = 0.018 [Figure 4a,b]). We noted no difference in low frequency between patients who underwent HRV biofeedback and those who received the sham intervention at any time point (Figure 4c,d). We did not note differences in frequency domain parameters of HRV between groups when assessed under paced breathing (Table S2).

Because we noted a difference in SDNN under normal breathing between main effect of groups at baseline in a regression model with positive interaction terms, we went on to perform a linear regression to investigate this difference solely at baseline, with adjustment for the same covariates. This analysis did not confirm a statistically significant between‐group difference at baseline but a nonsignificant trend remained (*ß* = −16.13, 95% CI −33.39, 1.12; *p* = 0.065).

### Functional outcome

**FIGURE 5:**
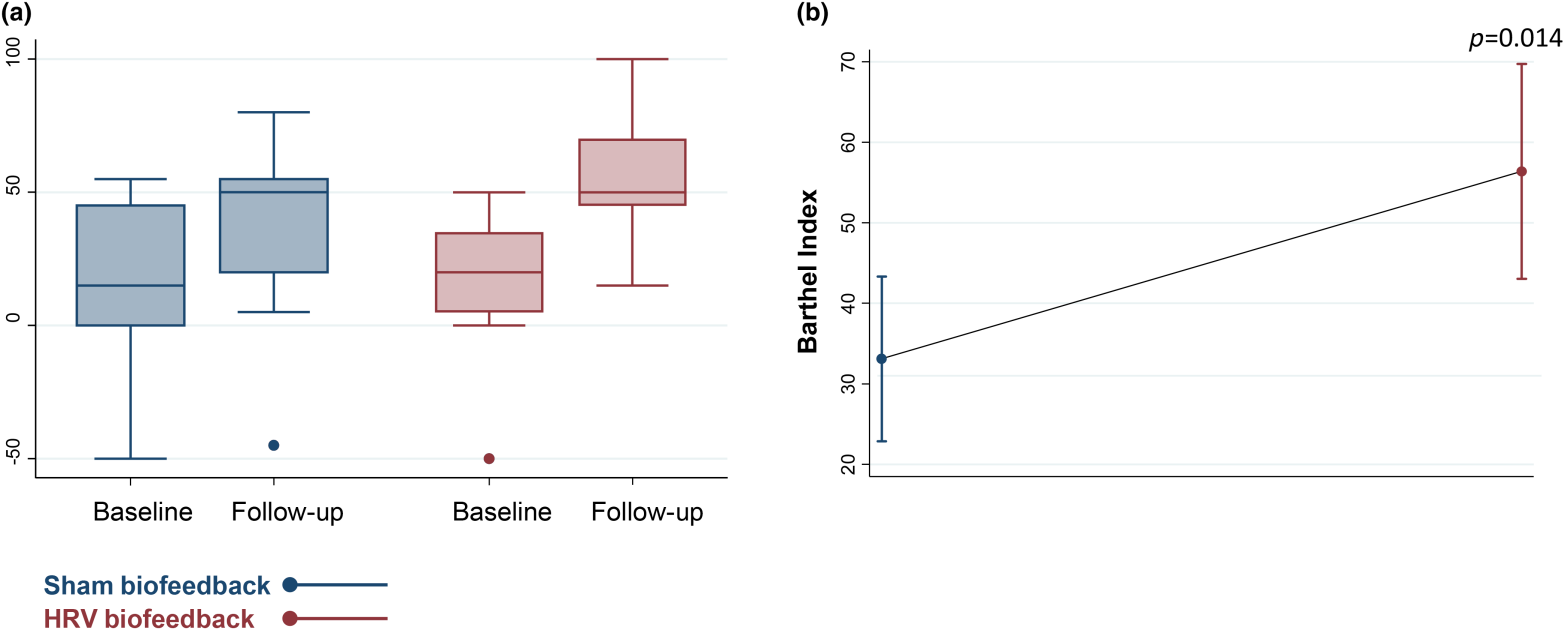
Functional disability at time of follow‐up. (a) Box plot of Barthel index values at the time of follow‐up depicting medians and interquartile ranges. (b) Linear prediction plot of disability at follow‐up assessed via Barthel index depicting predictive margins with 95% confidence intervals. The *p* value refers to between‐group comparison (HRV biofeedback vs. sham biofeedback).

Patients who underwent the HRV biofeedback intervention displayed a higher Barthel index, indicating lower disability at time of the 4‐week follow‐up compared to patients in the sham group (*ß* = 23.3, 95% CI 5.5, 41.1; *p* = 0.014) after adjustment for relevant covariates (Figure 5).

### Vasomotor autonomic function

Skin blood flow after sympathetic stimulation did not show any difference in patients who received HRV biofeedback training compared to those who underwent the sham intervention at any time point (Table S3).

### Sudomotor autonomic function

Sudomotor sympathetic skin response did not differ between patients who received HRV biofeedback training and those who received sham biofeedback at any time point (Table S4).

## DISCUSSION

The major findings of our study were, firstly, that a 2‐week protocol of daily HRV biofeedback sessions led to an improvement of neurocardiac function with increased parasympathetic measures of HRV in patients with CIP after sepsis undergoing inpatient rehabilitation. When assessed via SDNN, the primary outcome of our study, this increase in HRV post intervention was further sustained at the time of follow‐up, whereas patients in the sham group displayed a gradual decline. Secondly, sympathetic measures of autonomic functions did not differ between the two study groups, and thirdly, HRV biofeedback was associated with less severe functional disability at follow‐up. Taking these results together, parasympathetic modulation of heart function via HRV biofeedback seems to counteract progressive decline of HRV in CIP patients, associated with improved functional recovery.

Our observation of predominantly parasympathetic effects of HRV biofeedback on neurocardiac function is consistent with previous studies that applied the treatment in patients with coronary heart disease, acute ischemic stroke and psychiatric disorders, such as depression and panic disorder [8, 9, 16, 17, 18]. The majority of studies were conducted in ambulatory or hospital settings, potentially limiting comparability to our population of CIP patients undergoing comprehensive inpatient neurological rehabilitation. However, a feasibility trial of HRV biofeedback found an increase in SDNN in patients undergoing a standardized cardiac rehabilitation program in accordance with our observations in a population of patients with CIP after sepsis [19].

Our data support feasibility of HRV biofeedback as additive treatment in patients undergoing inpatient rehabilitation. Despite severe illness, our study patients completed the intervention except for one patient in the active study arm and two in the sham arm, who were lost to follow‐up. Neither of these patients dropped out for reasons related to the study or sham intervention.

In our study, HRV biofeedback was associated with improved neurocardiac function, with an elevation of parasympathetic measures of HRV. This observation, viewed in conjunction with the observed feasibility of the treatment, suggests HRV biofeedback might be a useful non‐invasive, non‐pharmacological treatment option to complement standard inpatient rehabilitation in this high‐risk population. The beneficial effect of HRV biofeedback on neurocardiac function remained statistically significant when adjusting our analysis for time from onset of acute illness. Therefore, the effect of HRV biofeedback on neurocardiac function seems not to be limited to specific phases of rehabilitation. However, the external validity of this finding warrants confirmation in a larger study population.

We observed a decline in time and frequency domain HRV parameters in patients who underwent sham biofeedback. This observation was unexpected but might be explained by the pathophysiology of CIP leading to continued cumulative damage of autonomic small nerve fibers. While recovery of somatic motoric function after CIP is often prolonged, with persisting deficits present in up to half of patients after 1 year, damage to autonomic small nerve fibers might be even more pronounced and long lasting since these fibers are characterized by thin or absence of myelin sheaths, contributing to their vulnerability [20, 21]. However, the exact pathophysiological mechanisms of damage to autonomic nerve fibers in patients with CIP remains poorly understood. Moreover, it should be noted that all of our patients received comprehensive rehabilitative and physical treatment focusing on improvement of motor and functional deficits, whereas no targeted treatment approach of autonomic impairment was part of the standard care regimen. Viewed in conjunction with previous investigations of the beneficial effects of physical therapy and early functional rehabilitation on clinical outcomes after CIP, our observation of improved neurocardiac function following HRV biofeedback suggests that adding this technique of autonomic neuromodulation to standard care might be useful in physical rehabilitation [22, 23, 24]. Furthermore, we observed a potentially favorable effect of HRV biofeedback on functional recovery, with less severe functional disability at the time of follow‐up. This observation supports a possible additive beneficial effect of HRV biofeedback on recovery from CIP after sepsis. However, we cannot comment on any causative association between elevation of HRV and less severe disability following HRV biofeedback due to the explorative nature of our study.

Interestingly, the beneficial effect of HRV biofeedback on neurocardiac function became apparent when HRV was assessed under normal but not under paced breathing conditions. The rationale of assessment of HRV under paced breathing is rooted in the elevation in parasympathetic HRV measure induced by metronomic breathing at six breathing cycles per minute, the same frequency used for HRV biofeedback. The absence of any effect of HRV biofeedback on HRV under paced breathing indicates that visual feedback is required in this severely ill population to allow effective execution of metronomic breathing. This is also consistent with the reporting of our study patients, where assessment under paced breathing was considered difficult in one third of patients, whereas the HRV biofeedback training itself was considered difficult in none of the patients.

Strengths of our study include its randomized controlled design as well as the detailed assessment of neurocardiac functions and sympathetic skin functions. Our study is limited by its explorative nature and by a small sample size. The latter likely explains the observed trend toward a difference between study arms in HRV at baseline despite randomization. However, our study showed a consistent beneficial effect of HRV biofeedback on neurocardiac function and functional recovery that remained significant across different predominantly parasympathetic measures of HRV after adjusting for clinically relevant covariates, supporting high internal validity. We cannot comment on the generalizability of our investigation; however, it provides pilot data for confirmatory follow‐up research. We did not perform electromyography at study entry, therefore, we could not discriminate between variants of CIP with and without overlapping myopathy in our population. However, the pathology whereby CIP may reduce HRV is likely primarily neurogenic and an equal distribution of overlapping myopathy between study groups can be assumed after randomization. Lastly, we did not perform any muscle or nerve biopsies. Hence, we cannot comment on the structural integrity of muscles and nerves in our study population.

In conclusion, in this explorative randomized sham‐controlled study in patients with CIP after sepsis, HRV biofeedback led to improved neurocardiac function with a predominant effect on the parasympathetic system and was associated with a lower degree of disability in patients with CIP. Our study provides pilot data for a confirmatory trial.

## AUTHOR CONTRIBUTIONS

**Annahita Sedghi:** Data curation; formal analysis; investigation; methodology; project administration; visualization; writing – original draft. **Christoph Bartels:** Data curation; investigation; resources; writing – review and editing; methodology; visualization. **Erik Simon:** Data curation; methodology; writing – review and editing. **Florian Krause:** Methodology; writing – review and editing. **Martin Arndt:** Methodology; writing – review and editing. **Stefan Zsigri:** Methodology; writing – review and editing. **Kristian Barlinn:** Project administration; writing – review and editing. **Ulf Bodechtel:** Conceptualization; funding acquisition; project administration; supervision; resources; writing – review and editing. **Ana Isabel Penzlin:** Conceptualization; funding acquisition; methodology; project administration; writing – review and editing. **Timo Siepmann:** Conceptualization; methodology; funding acquisition; project administration; resources; supervision; visualization; writing – original draft; writing – review and editing.

## FUNDING INFORMATION

This study was funded by a grant of the Kurt Goldstein Institut. Open access funding was covered by Project DEAL.

## CONFLICT OF INTEREST STATEMENT

The authors declare no commercial or financial relationships that could be construed as a potential conflict of interest.

## Supporting information


Data S1:

Supplementary Information S1:

Supplementary Information S2:

Table S1:

Table S2:

Table S3:

Table S4:

## Data Availability

The data that support the findings of this study are available from the corresponding author upon reasonable request.
