# Supplementary material for: Heart rate variability biofeedback for critical illness polyneuropathy: a randomized sham‐controlled study
Source: Eur J Neurol. 2024 Oct 18;31(12):e16512. doi: 10.1111/ene.16512 (PMC11554868; doi:10.1111/ene.16512)
Supplement: Supplementary file 2 — Supplementary Information S1: [file ENE-31-e16512-s004.pdf]

Supplementary Information S1 Nerve conduction studies and muscle strength at admission

Nerve conduction studies

Study Patient 1

	Motor						Sensory			
	Distal motor latency (m/s)		Compound muscle action potential (mV)		Motor nerve conduction velocity (m/s)		Sensory nerve action potential (µV)		Sensory nerve conduction velocity (m/s)	
	ULV	Value	LLV	Value	LLV	Value	LLV	Value	LLV	Value
**N. ulnaris (right)**	3.5	2.5	8.0	10.6	50 – 55	47.4	15.0	20.6	50.0	47.4
**N. tibialis (right)**	6.0	2.9	8.0	2.7	40.0	32.7	-	-	-	-
**N. tibialis (left)**	6.0	3.8	8.0	1.6	40.0	35.5	-	-	-	-
**N. peroneus (right)**	No potential detectable									
**N. peroneus (left)**	No potential detectable									
**N. suralis (right)**	No potential detectable									
**N. suralis (left)**	No potential detectable									

Study Patient 2

	Motor						Sensory			
	Distal motor latency (m/s)		Compound muscle action potential (mV)		Motor nerve conduction velocity (m/s)		Sensory nerve action potential (µV)		Sensory nerve conduction velocity (m/s)	
	ULV	Value	LLV	Value	LLV	Value	LLV	Value	LLV	Value
**N. medianus (right)**	4.2	5.63	8.0	3.7	48 – 55	53.7	12.0	9.8	45.0	32.2
**N. peroneus (left)**	5.6	4.12	5.0	2.1	42.0	37.1	10.0	/	40.0	/
**N. suralis (right)**	-	-	-	-	-	-	10.0	1.44	42.0	39.5
**N. tibialis (right)**	6.0	3.68	8.0	2.7	40.0	33.4	-	-	-	-

Study Patient 3

	Motor						Sensory			
	Distal motor latency (m/s)		Compound muscle action potential (mV)		Motor nerve conduction velocity (m/s)		Sensory nerve action potential (µV)		Sensory nerve conduction velocity (m/s)	
	ULV	Value	LLV	Value	LLV	Value	LLV	Value	LLV	Value
**N. medianus (right)**	4.2	3.3	8.0	11.5	48 – 55	47	8.0	7.9	48.0	57
**N. tibialis (right)**	6.0	4.1	8.0	2.5	40.0	35	-	-	-	-
**N. peroneus (left)**	5.6	5.0	5.0	6.4	40.0	31	10.0	/	40.0	/
**N. suralis (right)**	-	-	-	-	-	-	No potential detectable			

Study Patient 4

	Motor						Sensory			
	Distal motor latency (m/s)		Compound muscle action potential (mV)		Motor nerve conduction velocity (m/s)		Sensory nerve action potential (µV)		Sensory nerve conduction velocity (m/s)	
	ULV	Value	LLV	Value	LLV	Value	LLV	Value	LLV	Value
**N. ulnaris (right)**	3.5	2.9	8.0	14.9	50 – 55	51.0	15.0	20.1	50.0	45.0
**N. ulnaris (left)**	3.5	3.0	8.0	8.2	50 – 55	53.3	15.0	14.0	50.0	44.0
**N. tibialis (right)**	No potential detectable									
**N. tibialis (left)**	No potential detectable									
**N. peroneus (right)**	No potential detectable									
**N. peroneus (left)**	No potential detectable									
**N. suralis (right)**	No potential detectable									
**N. suralis (left)**	No potential detectable									

Study Patient 5

	Motor						Sensory			
	Distal motor latency (m/s)		Compound muscle action potential (mV)		Motor nerve conduction velocity (m/s)		Sensory nerve action potential (µV)		Sensory nerve conduction velocity (m/s)	
	ULV	IST	LLV	IST	LLV	IST	LLV	IST	LLV	IST
**N. medianus (left)**	4.2	4.4	8.0	3.7	48 – 55	40	8.0	13.8	48.0	39
**N. tibialis (right)**	No potential detectable						-	-	-	-
**N. peroneus (left)**	No potential detectable						10.0	/	40.0	/
**N. suralis (right)**	-	-	-	-	-	-	4.0	/	40.0	/

Study Patient 6

	Motor						Sensory			
	Distal motor latency (m/s)		Compound muscle action potential (mV)		Motor nerve conduction velocity (m/s)		Sensory nerve action potential (µV)		Sensory nerve conduction velocity (m/s)	
	ULV	Value	LLV	Value	LLV	Value	LLV	Value	LLV	Value
**N. ulnaris (right)**	3.5	2.6	8.0	11.0	50 – 55	50	15.0	29	50.0	49
**N. tibialis (right)**	6.0	4.9	8.0	13.7	40.0	31	-	-	-	-
**N. tibialis (left)**	6.0	4.5	8.0	12.6	40.0	36	-	-	-	-
**N. peroneus (right)**	No potential detectable						10.0	/	40.0	/
**N. peroneus (left)**	5.6	5.75	5.0	0.28	42.0	28	10.0	/	40.0	/
**N. suralis (right)**	-	-	-	-	-	-	10.0	9.7	42.0	35
**N. suralis (left)**	-	-	-	-	-	-	10.0	6.0	42.0	31

Study Patient 7

	Motor						Sensory			
	Distal motor latency (m/s)		Compound muscle action potential (mV)		Motor nerve conduction velocity (m/s)		Sensory nerve action potential (µV)		Sensory nerve conduction velocity (m/s)	
	ULV	Value	LLV	Value	LLV	Value	LLV	Value	LLV	Value
**N. ulnaris (right)**	3.5	2.3	8.0	15.0	50 – 55	51	15.0	6.0	50.0	57
**N. tibialis (right)**	6.0	3.5	8.0	12.8	40.0	43.3	-	-	-	-
**N. tibialis (left)**	6.0	3.8	8.0	16.4	40.0	43.6	-	-	-	-
**N. peroneus (right)**	5.6	3.6	5.0	3.4	42.0	39.4	10.0	/	40.0	/
**N. peroneus (left)**	5.6	3.8	5.0	5.5	42.0	41	10.0	/	40.0	/
**N. suralis (right)**	-	-	-	-	-	-	10.0	7.2	42.0	55.3
**N. suralis (left)**	-	-	-	-	-	-	10.0	6.0	42.0	38

Study Patient 8

	Motor						Sensory			
	Distal motor latency (m/s)		Compound muscle action potential (mV)		Motor nerve conduction velocity (m/s)		Sensory nerve action potential (µV)		Sensory nerve conduction velocity (m/s)	
	ULV	Value	LLV	Value	LLV	Value	LLV	Value	LLV	Value
**N. medianus (right)**	4.2	3.7	8.0	6.8	48 – 55	29	8.0	n.a.	48.0	n.a.
**N. tibialis (right)**	6.0	4.0	8.0	1.4	40.0	36	-	-	-	-
**N. peroneus (left)**	5.6	3.7	5.0	0.3	40.0	35	10.0	/	40.0	/
**N. suralis (right)**	-	-	-	-	-	-	4.0	4.0	40.0	38

Study Patient 9

	Motor						Sensory			
	Distal motor latency (m/s)		Compound muscle action potential (mV)		Motor nerve conduction velocity (m/s)		Sensory nerve action potential (µV)		Sensory nerve conduction velocity (m/s)	
	ULV	Value	LLV	Value	LLV	Value	LLV	Value	LLV	Value
**N. ulnaris (right)**	3.5	3.62	8.0	12.6	50 – 55	50.2	15.0	10.2	50.0	46
**N. tibialis (right)**	6.0	5.6	8.0	6.8	40.0	41	-	-	-	-
**N. tibialis (left)**	6.0	3.2	8.0	4.2	40.0	38	-	-	-	-
**N. peroneus (right)**	5.6	4.2	5.0	7.0	42.0	39	10.0	/	40.0	/
**N. peroneus (left)**	5.6	2.8	5.0	3.1	42.0	36.4	10.0	/	40.0	/
**N. suralis (right)**	-	-	-	-	-	-	10.0	6.0	42.0	33
**N. suralis (left)**	-	-	-	-	-	-	10.0	3.0	42.0	31

Study Patient 10

	Motor						Sensory			
	Distal motor latency (m/s)		Compound muscle action potential (mV)		Motor nerve conduction velocity (m/s)		Sensory nerve action potential (µV)		Sensory nerve conduction velocity (m/s)	
	ULV	Value	LLV	Value	LLV	Value	LLV	Value	LLV	Value
**N. ulnaris (right)**	3.5	3.1	8.0	9.9	50 – 55	55.6	15.0	19.0	50.0	52.7
**N. tibialis (right)**	6.0	3.0	8.0	4.4	40.0	45.8	-	-	-	-
**N. tibialis (left)**	6.0	4.2	8.0	2.3	40.0	56.2	-	-	-	-
**N. peroneus (right)**	5.6	4.5	5.0	0.3	42.0	53.8	10.0	/	40.0	/
**N. peroneus (left)**	No potential detectable						10.0	/	40.0	/
**N. suralis (right)**	-	-	-	-	-	-	10.0	7.9	42.0	51.5
**N. suralis (left)**	-	-	-	-	-	-	10.0	4.9	42.0	50.5

Study Patient 11

	Motor						Sensory			
	Distal motor latency (m/s)		Compound muscle action potential (mV)		Motor nerve conduction velocity (m/s)		Sensory nerve action potential (µV)		Sensory nerve conduction velocity (m/s)	
	ULV	Value	LLV	Value	LLV	Value	LLV	Value	LLV	Value
**N. ulnaris (right)**	3.5	8.1	8.0	0.1	50 – 55	19.0	15.0	n.a.	50.0	n.a.
**N. ulnaris (left)**	3.5	8.4	8.0	0.1	50 – 55	15.0	15.0	n.a.	50.0	n.a.
**N. tibialis (right)**	No potential detectable						-	-	-	-
**N. tibialis (left)**	No potential detectable					-	-	-	-	-
**N. peroneus (right)**	No potential detectable					-	10.0	/	40.0	/
**N. peroneus (left)**	No potential detectable					-	10.0	/	40.0	/
**N. suralis (right)**	-	-	-	-	-	-	10.0	n.a.	42.0	n.a.
**N. suralis (left)**	-	-	-	-	-	-	10.0	n.a.	42.0	n.a.

Study Patient 12

	Motor						Sensory			
	Distal motor latency (m/s)		Compound muscle action potential (mV)		Motor nerve conduction velocity (m/s)		Sensory nerve action potential (µV)		Sensory nerve conduction velocity (m/s)	
	ULV	Value	LLV	Value	LLV	Value	LLV	Value	LLV	Value
**N. ulnaris (right)**	3.5	3.4	8.0	12	50 – 55	39	15.0		50.0	
**N. tibialis (right)**	6.0	4.4	8.0	5.2	40.0	33	-	-	-	-
**N. tibialis (left)**	6.0	4.3	8.0	4.2	40.0	33	-	-	-	-
**N. peroneus (right)**	5.6	n.a.	5.0	n.a.	42.0	n.a.	10.0	/	40.0	/
**N. peroneus (left)**	5.6	6.5	5.0	0.7	42.0	33	10.0	/	40.0	/
**N. suralis (right)**	-	-	-	-	-	-	10.0	n.a.	42.0	n.a.
**N. suralis (left)**	-	-	-	-	-	-	10.0	6.0	42.0	30

Study Patient 13

	Motor						Sensory			
	Distal motor latency (m/s)		Compound muscle action potential (mV)		Motor nerve conduction velocity (m/s)		Sensory nerve action potential (µV)		Sensory nerve conduction velocity (m/s)	
	ULV	Value	LLV	Value	LLV	Value	LLV	Value	LLV	Value
**N. ulnaris (right)**	3.5	3.9	8.0	8	50 – 55	41	15.0	5	50.0	42
**N. tibialis (right)**	6.0	n.a.	8.0	n.a.	40.0	-	-	-	-	-
**N. tibialis (left)**	6.0	n.a.	8.0	n.a.	40.0	-	-	-	-	-
**N. peroneus (right)**	5.6	n.a.	5.0	n.a.	42.0	-	10.0	/	40.0	/
**N. peroneus (left)**	5.6	n.a.	5.0	n.a.	42.0	-	10.0	/	40.0	/
**N. suralis (right)**	-	-	-	-	-	-	10.0	-	42.0	-
**N. suralis (left)**	-	-	-	-	-	-	10.0	-	42.0	-

Study Patient 14

	Motor						Sensory			
	Distal motor latency (m/s)		Compound muscle action potential (mV)		Motor nerve conduction velocity (m/s)		Sensory nerve action potential (µV)		Sensory nerve conduction velocity (m/s)	
	ULV	Value	LLV	Value	LLV	Value	LLV	Value	LLV	Value
**N. ulnaris (right)**	3.5	4.1	8.0	10.4	50 – 55	62.1	15.0	2.3	50.0	38.0
**N. tibialis (right)**	6.0	5.6	8.0	3.6	40.0	34.0	-	-	-	-
**N. tibialis (left)**	6.0	5.4	8.0	1.4	40.0	35.5	-	-	-	-
**N. peroneus (right)**	5.6	4.1	5.0	0.8	42.0	31.0	10.0	/	40.0	/
**N. peroneus (left)**	5.6	4.5	5.0	0.2	42.0	30.0	10.0	/	40.0	/
**N. suralis (right)**	-	-	-	-	-	-	10.0	2.7	42.0	38.0
**N. suralis (left)**	-	-	-	-	-	-	10.0	2.4	42.0	37.5

Study Patient 15

	Motor						Sensory			
	Distal motor latency (m/s)		Compound muscle action potential (mV)		Motor nerve conduction velocity (m/s)		Sensory nerve action potential (µV)		Sensory nerve conduction velocity (m/s)	
	ULV	Value	LLV	Value	LLV	Value	LLV	Value	LLV	Value
**N. medianus (right)**	4.2	5.5	8.0	3.2	48 – 55	32	8.0	0.8	48.0	28
**N. tibialis (right)**	6.0	3.6	8.0	3.1	40.0	39	-	-	-	-
**N. peroneus (left)**	5.6	4.4	4.0	2.1	40.0	47	10.0	/	40.0	/
**N. suralis (right)**	-	-	-	-	-	-	4.0	3.7	40.0	45

Study Patient 16

	Motor						Sensory			
	Distal motor latency (m/s)		Compound muscle action potential (mV)		Motor nerve conduction velocity (m/s)		Sensory nerve action potential (µV)		Sensory nerve conduction velocity (m/s)	
	ULV	Value	LLV	Value	LLV	Value	LLV	Value	LLV	Value
**N. medianus (right)**	4.2	3.1	8.0	3.6	48 – 55	49	8.0	15.8	48.0	53.1
**N. tibialis (right)**	6.0	3.3	8.0	6.8	40.0	43	-	-	-	-
**N. peroneus (left)**	5.6	4.2	4.0	0.25	40.0	49.4	10.0	/	40.0	/
**N. suralis (right)**	-	-	-	-	-	-	4.0	9.5	40.0	55.3

Study Patient 17

	Motor						Sensory			
	Distal motor latency (m/s)		Compound muscle action potential (mV)		Motor nerve conduction velocity (m/s)		Sensory nerve action potential (µV)		Sensory nerve conduction velocity (m/s)	
	ULV	Value	LLV	Value	LLV	Value	LLV	Value	LLV	Value
**N. medianus (right)**	4.2	3.6	8.0	4.5	48 – 55	47	8.0	19.1	48.0	55
**N. medianus (left)**	4.2	3.3	8.0	3.9	48 – 55	48	8.0	15.6	48.0	53
**N. tibialis (right)**	6.0	5.4	8.0	0.3	40.0	37	-	-	-	-
**N. peroneus (left)**	No potential detectable						10.0	/	40.0	/
**N. suralis (left)**	-	-	-	-	-	-	No potential detectable			

Study Patient 18

	Motor						Sensory			
	Distal motor latency (m/s)		Compound muscle action potential (mV)		Motor nerve conduction velocity (m/s)		Sensory nerve action potential (µV)		Sensory nerve conduction velocity (m/s)	
	ULV	Value	LLV	Value	LLV	Value	LLV	Value	LLV	Value
**N. medianus (right)**	4.2	4.34	8.0	6.6	48 – 55	42.9	12.0	4.9	45.0	47.6
**N. peroneus (left)**	5.6	3.85	5.0	1.25	42.0	34.8	10.0	/	40.0	/
**N. suralis (right)**	-	-	-	-	-	-	10.0	3.7	42.0	42.0
**N. tibialis (right)**	6.0	3.79	8.0	2.0	40.0	36.4	-	-	-	-

Study Patient 19

	Motor						Sensory			
	Distal motor latency (m/s)		Compound muscle action potential (mV)		Motor nerve conduction velocity (m/s)		Sensory nerve action potential (µV)		Sensory nerve conduction velocity (m/s)	
	ULV	Value	LLV	Value	LLV	Value	LLV	Value	LLV	Value
**N. ulnaris (right)**	3.5	2.7	8.0	8.3	50 – 55	51.0	15.0	17.5	50.0	42.7
**N. tibialis (right)**	6.0	3.8	8.0	7.4	40.0	36.0	-	-	-	-
**N. tibialis (left)**	6.0	4.2	8.0	5.7	40.0	34.0	-	-	-	-
**N. peroneus (right)**	5.6	4.7	5.0	0.4	42.0	31.0	10.0	/	40.0	/
**N. peroneus (left)**	5.6	4.8	5.0	0.9	42.0	32.0	10.0	/	40.0	/
**N. suralis (right)**	-	-	-	-	-	-	10.0	9.0	42.0	35.0
**N. suralis (left)**	-	-	-	-	-	-	10.0	9.3	42.0	39.0

Study Patient 20

	Motor						Sensory			
	Distal motor latency (m/s)		Compound muscle action potential (mV)		Motor nerve conduction velocity (m/s)		Sensory nerve action potential (µV)		Sensory nerve conduction velocity (m/s)	
	ULV	Value	LLV	Value	LLV	Value	LLV	Value	LLV	Value
**N. ulnaris (right)**	3.5	1.72	8.0	9.5	50 – 55	53.3	15.0	18.4	50.0	54.3
**N. tibialis (right)**	6.0	2.63	8.0	8.9	40.0	54.6	-	-	-	-
**N. tibialis (left)**	6.0	2.93	8.0	5.1	40.0	49.5	-	-	-	-
**N. peroneus (right)**	5.6	3.53	5.0	2.2	42.0	42.2	10.0	/	40.0	/
**N. peroneus (left)**	5.6	3.42	5.0	1.82	42.0	39.7	10.0	/	40.0	/
**N. suralis (right)**	-	-	-	-	-	-	10.0	2.2	42.0	42.2
**N. suralis (left)**	-	-	-	-	-	-	10.0	2.9	42.0	58.5

Study Patient 21

	Motor						Sensory			
	Distal motor latency (m/s)		Compound muscle action potential (mV)		Motor nerve conduction velocity (m/s)		Sensory nerve action potential (µV)		Sensory nerve conduction velocity (m/s)	
	ULV	Value	LLV	Value	LLV	Value	LLV	Value	LLV	Value
**N. ulnaris (right)**	3.5	3.0	8.0	14	50 – 55	52	15.0	21	50.0	53
**N. tibialis (right)**	6.0	4.5	8.0	16.5	40.0	48	-	-	-	-
**N. tibialis (left)**	6.0	4.4	8.0	13	40.0	42	-	-	-	-
**N. peroneus (right)**	5.6	4.0	5.0	7.8	42.0	42	10.0	/	40.0	/
**N. peroneus (left)**	5.6	5.8	5.0	1.4	42.0	44	10.0	/	40.0	/
**N. suralis (right)**	No potential detectable									
**N. suralis (left)**	No potential detectable									

Study Patient 22

	Motor						Sensory			
	Distal motor latency (m/s)		Compound muscle action potential (mV)		Motor nerve conduction velocity (m/s)		Sensory nerve action potential (µV)		Sensory nerve conduction velocity (m/s)	
	ULV	Value	LLV	Value	LLV	Value	LLV	Value	LLV	Value
**N. ulnaris (right)**	3.5	2.3	8.0	7	50 – 55	45	15.0	8.2	50.0	52
**N. tibialis (right)**	6.0	3.1	8.0	9	40.0	36	-	-	-	-
**N. tibialis (left)**	6.0	3.7	8.0	3.5	40.0	38	-	-	-	-
**N. peroneus (right)**	5.6	2.8	5.0	0.4	42.0	27	10.0	/	40.0	/
**N. peroneus (left)**	No potential detectable									
**N. suralis (right)**	-	-	-	-	-	-	10.0	1.7	42.0	33.2
**N. suralis (left)**	No potential detectable									

Study Patient 23

	Motor						Sensory			
	Distal motor latency (m/s)		Compound muscle action potential (mV)		Motor nerve conduction velocity (m/s)		Sensory nerve action potential (µV)		Sensory nerve conduction velocity (m/s)	
	ULV	Value	LLV	Value	LLV	Value	LLV	Value	LLV	Value
**N. ulnaris (right)**	3.5	1.7	8.0	11.4	50 – 55	43.5	15.0	12	50.0	49
**N. tibialis (right)**	6.0	2.9	8.0	10	40.0	43	-	-	-	-
**N. tibialis (left)**	6.0	2.4	8.0	9.6	40.0	40	-	-	-	-
**N. peroneus (right)**	5.6	3.4	5.0	3	42.0	46	10.0	/	40.0	/
**N. peroneus (left)**	5.6	3.3	5.0	1.2	42.0	42	10.0	/	40.0	/
**N. suralis (right)**	-	-	-	-	-	-	10.0	4.2	42.0	44
**N. suralis (left)**	-	-	-	-	-	-	10.0	8	42.0	43

Study Patient 24

	Motor						Sensory			
	Distal motor latency (m/s)		Compound muscle action potential (mV)		Motor nerve conduction velocity (m/s)		Sensory nerve action potential (µV)		Sensory nerve conduction velocity (m/s)	
	ULV	Value	LLV	Value	LLV	Value	LLV	Value	LLV	Value
**N. medianus (right)**	4.2	3.54	8.0	2.9	48 – 55	51.1	12.0	3.7	45.0	52.0
**N. peroneus (left)**	5.6	5.38	5.0	0.78	42.0	41.4	10.0	/	40.0	/
**N. suralis (right)**	-	-	-	-	-	-	10.0	2.3	42.0	31.7
**N. tibialis (right)**	6.0	5.07	8.0	2.7	40.0	36.7	-	-	-	-

Study Patient 25

	Motor						Sensory			
	Distal motor latency (m/s)		Compound muscle action potential (mV)		Motor nerve conduction velocity (m/s)		Sensory nerve action potential (µV)		Sensory nerve conduction velocity (m/s)	
	ULV	Value	LLV	Value	LLV	Value	LLV	Value	LLV	Value
**N. ulnaris (right)**	3.5	2.0	8.0	10.0	50 – 55	56.0	15.0	6.5	50.0	50.0
**N. tibialis (right)**	6.0	3.0	8.0	15.0	40.0	47.0	-	-	-	-
**N. tibialis (left)**	6.0	3.9	8.0	0.24	40.0	42.0	-	-	-	-
**N. peroneus (right)**	5.6	2.8	5.0	2.9	42.0	42.0	10.0	/	40.0	/
**N. peroneus (left)**	No potential detectable									
**N. suralis (right)**	-	-	-	-	-	-	10.0	4.4	42.0	48.0
**N. suralis (left)**	No potential detectable									

Study Patient 26

	Motor						Sensory			
	Distal motor latency (m/s)		Compound muscle action potential (mV)		Motor nerve conduction velocity (m/s)		Sensory nerve action potential (µV)		Sensory nerve conduction velocity (m/s)	
	ULV	Value	LLV	Value	LLV	Value	LLV	Value	LLV	Value
**N. ulnaris (right)**	3.5	3.0	8.0	8.0	50 – 55	75.0	15.0	29.0	50.0	42.5
**N. tibialis (right)**	6.0	4.5	8.0	4.0	40.0	35.0	-	-	-	-
**N. tibialis (left)**	6.0	4.5	8.0	7.0	40.0	36.0	-	-	-	-
**N. peroneus (right)**	No potential detectable									
**N. peroneus (left)**	5.6	5.1	5.0	0.4	42.0	38.0	10.0	/	40.0	/
**N. suralis (right)**	-	-	-	-	-	-	10.0	1.0	42.0	34.5
**N. suralis (left)**	-	-	-	-	-	-	10.0	2.2	42.0	38.0

Study Patient 27

	Motor						Sensory			
	Distal motor latency (m/s)		Compound muscle action potential (mV)		Motor nerve conduction velocity (m/s)		Sensory nerve action potential (µV)		Sensory nerve conduction velocity (m/s)	
	ULV	Value	LLV	Value	LLV	Value	LLV	Value	LLV	Value
**N. tibialis (left)**	6.0	4.0	8.0	0.9	40.0	39.5	-	-	-	-
**N. peroneus (left)**	No potential detectable						10.0	/	40.0	/
**N. suralis (left)**	No potential detectable									
**N. femoralis (left)**	No potential detectable									

Study Patient 28

	Motor						Sensory			
	Distal motor latency (m/s)		Compound muscle action potential (mV)		Motor nerve conduction velocity (m/s)		Sensory nerve action potential (µV)		Sensory nerve conduction velocity (m/s)	
	**ULV**	**Value**	**LLV**	**Value**	**LLV**	**Value**	**LLV**	**Value**	**LLV**	**Value**
**N. medianus (right)**	4.2	2.84	8.0	1.98	48 – 55	36.0	12.0	9.1	45.0	46.3
**N. peroneus (right)**	5.6	5.26	5.0	0.10	42.0	32.9	10.0	/	40.0	/
**N. suralis (right)**	-	-	-	-	-	-	10.0	n.a.	42.0	n.a.
**N. tibialis (left)**	No potential detectable						-	-	-	-

Study Patient 29

	Motor						Sensory			
	Distal motor latency (m/s)		Compound muscle action potential (mV)		Motor nerve conduction velocity (m/s)		Sensory nerve action potential (µV)		Sensory nerve conduction velocity (m/s)	
	**ULV**	**Value**	**LLV**	**Value**	**LLV**	**Value**	**LLV**	**Value**	**LLV**	**Value**
**N. medianus (right)**	4.2	4.30	8.0	7.0	48 – 55	53.2	12.0	17.8	45.0	43.6
**N. peroneus (left)**	5.6	4.22	5.0	0.13	42.0	32.0	10.0	/	40.0	/
**N. suralis (right)**	-	-	-	-	-	-	10.0	9.8	42.0	59.5
**N. tibialis (right)**	6.0	3.03	8.0	10.4	40.0	44.5	-	-	-	-

Study Patient 30

	Motor						Sensory			
	Distal motor latency (m/s)		Compound muscle action potential (mV)		Motor nerve conduction velocity (m/s)		Sensory nerve action potential (µV)		Sensory nerve conduction velocity (m/s)	
	**ULV**	**Value**	**LLV**	**Value**	**LLV**	**Value**	**LLV**	**Value**	**LLV**	**Value**
**N. ulnaris (left)**	3.5	3.6	8.0	10	50 – 55	54	15.0	12	50.0	39
**N. tibialis (right)**	6.0	4	8.0	5.5	40.0	39.5	-	-	-	-
**N. tibialis (left)**	6.0	4.7	8.0	3	40.0	42	-	-	-	-
**N. peroneus (right)**	5.6	3.3	5.0	1.2	42.0	41.5	10.0	/	40.0	/
**N. peroneus (left)**	5.6	3.5	5.0	1	42.0	42	10.0	/	40.0	/
**N. suralis (right)**	-	-	-	-	-	-	10.0	3	42.0	55
**N. suralis (left)**	-	-	-	-	-	-	10.0	2.5	42.0	51

ULV, Upper limit value; LLV, lower limit value; n.m.=not measured

Study Patient 30

	Motor						Sensory			
	Distal motor latency (m/s)		Compound muscle action potential (mV)		Motor nerve conduction velocity (m/s)		Sensory nerve action potential (µV)		Sensory nerve conduction velocity (m/s)	
	**ULV**	**Value**	**LLV**	**Value**	**LLV**	**Value**	**LLV**	**Value**	**LLV**	**Value**
**N. ulnaris (left)**	3.5	3.6	8.0	10	50 – 55	54	15.0	12	50.0	39
**N. tibialis (right)**	6.0	4	8.0	5.5	40.0	39.5	-	-	-	-
**N. tibialis (left)**	6.0	4.7	8.0	3	40.0	42	-	-	-	-
**N. peroneus (right)**	5.6	3.3	5.0	1.2	42.0	41.5	10.0	/	40.0	/
**N. peroneus (left)**	5.6	3.5	5.0	1	42.0	42	10.0	/	40.0	/
**N. suralis (right)**	-	-	-	-	-	-	10.0	3	42.0	55
**N. suralis (left)**	-	-	-	-	-	-	10.0	2.5	42.0	51

Muscle strength at admission

(Values according to Medical Research Council Scale for Muscle Strength)

Patient	Muscle Strength: Arms proximal (right)	Muscle Strength: Arms proximal (left)	Muscle Strength: Arms dIstal (right)	Muscle Strength: Arms distal (left)	Muscle Strength: untere Extremtitäten proximal (right)	Muscle Strength: Legs proximal (left)	Muscle Strength: Legs distal (right)	Muscle Strength: Legs distal (left)
1	3	3	4	4	3	4	3	4
2	4	4	4	4	3	3	3	3
3	4	4	4	3	3	4	4	4
4	2	1	1	1	2	1	1	1
5	4	4	4	4	4	4	4	4
6	4	4	3	3	2	2	3	3
7	4	4	4	4	2	2	3	3
8	4	4	4	4	4	4	4	4
9	4	4	4	4	3	3	3	3
10	4	4	4	4	2	3	3	3
11	4	4	4	4	3	3	3	3
12	4	4	4	4	3	3	4	4
13	4	4	4	4	2	3	2	3
14	4	4	4	4	3	3	2	2
15	4	4	4	4	4	4	4	4
16	4	4	4	4	3	3	4	4
17	4	4	4	4	4	4	3	3
18	2	3	2	3	2	3	2	3
19	4	4	4	4	2	2	2	2
20	4	4	4	4	3	3	2	2
21	3	3	4	4	3	3	4	4
22	5	5	4	4	4	4	2	2
23	4	4	4	4	3	3	3	3
24	4	4	4	4	4	4	4	4
25	3	0	3	0	2	0	2	0
26	4	4	4	4	3	3	4	4
27	4	4	3	3	3	3	2	0
28	4	4	4	4	3	3	3	3
29	4	4	4	4	3	3	3	3
30	3	3	3	3	3	3	3	3
