# Supplementary material for: Heart rate variability biofeedback for critical illness polyneuropathy: a randomized sham‐controlled study
Source: Eur J Neurol. 2024 Oct 18;31(12):e16512. doi: 10.1111/ene.16512 (PMC11554868; doi:10.1111/ene.16512)
Supplement: Supplementary file 3 — Supplementary Information S2: [file ENE-31-e16512-s006.pdf]

Supplementary Information S2 Study population

Mode of admission

Patients were transferred to the rehabilitation clinic from regular acute wards (n=11), intermediate care wards (n=1) as well as intensive care units (n=18). Twelve patients had a feeding nasal tube and one had a tracheal tube at the time of admission.

Medical history and diagnosis

Four patients had a past history of stroke. None of the patients had a history of autonomic diabetic neuropathy. None of the patients had cardiac arrhythmia interfering with HRV assessment. All patients had a diagnosis of CIP at the time of admission. Electroneurography studies were performed during in-patient rehabilitation to confirm the diagnosis before study entry. All patients had a diagnosis of CIP at the time of study entry.

Time to intervention

The median time between admission and first day of HRV or sham biofeedback was 80 [5-106, IQR].
