# Supplementary material for: Heart rate variability biofeedback for critical illness polyneuropathy: a randomized sham‐controlled study
Source: Eur J Neurol. 2024 Oct 18;31(12):e16512. doi: 10.1111/ene.16512 (PMC11554868; doi:10.1111/ene.16512)
Supplement: Supplementary file 4 — Table S1: [file ENE-31-e16512-s007.pdf]

Supplementary Table S1 Time domain analysis of HRV under paced breathing

	**Baseline**	**Post-intervention**	**Follow-up**
HRV biofeedback			
**SDNN**	17.16 [10.22, 30.92]	36.09 [16.81,43.73]	17.67 [10.3, 34.95]
**RMSSD**	9.24 [4.24, 29.8]	20.23 [9.02, 60.23]	9.15 [5.95, 15.02]
Sham biofeedback			
**SDNN**	16.72 [9.74,41.14]	27.52 [12.08,44.15]	16.09 [9.70,23.47]
**RMSSD**	13.82 [4.88,57.9]	11.07 [5.98,42.32]	11.28 [5.78,28.93]

Legend to Supplementary Table S1

All values in median [interquartile range]. Interaction effects between group and time points of measurement were not significant for SDNN and RMSSD ($p>0.05$). Abbreviations: SDNN, standard deviation of normal beat-to-beat intervals; RMSSD, root mean square of successive RR interval differences
