# Supplementary material for: Heart rate variability biofeedback for critical illness polyneuropathy: a randomized sham‐controlled study
Source: Eur J Neurol. 2024 Oct 18;31(12):e16512. doi: 10.1111/ene.16512 (PMC11554868; doi:10.1111/ene.16512)
Supplement: Supplementary file 5 — Table S2: [file ENE-31-e16512-s005.pdf]

Supplementary Table S2 Spectral analysis of HRV under paced breathing

	**Baseline**	**Post-intervention**	**Follow-up**
HRV biofeedback			
**LF**	41.69 [20.18,113.6]	168.1 [36.59,820.5]	117 [22.29,439.7]
**HF**	20.79 [4.09,191.5]	131.2 [19.14,521.8]	32.8 [8.90,97.18]
Sham biofeedback			
**LF**	26.36 [6.56,174.8]	24.99 [15.71,246.6]	34.22 [9.59,89.68]
**HF**	30.76 [6.53,155]	87.51 [21.69,790.7]	29.18 [7.83,170.5]

Legend to Supplementary Table S1

All values in median [interquartile range]. Interaction effects between group and time points of measurement were not significant for LF and HF under paced breathing ($p>0.05$).

Abbreviations: LF, low frequency; HF, high frequency
