# Supplementary material for: Heart rate variability biofeedback for critical illness polyneuropathy: a randomized sham‐controlled study
Source: Eur J Neurol. 2024 Oct 18;31(12):e16512. doi: 10.1111/ene.16512 (PMC11554868; doi:10.1111/ene.16512)
Supplement: Supplementary file 6 — Table S3: [file ENE-31-e16512-s002.pdf]

Supplementary Table S3 Skin blood flow response after sympathetic stimulation

	Baseline	Post-intervention	Follow-up
HRV biofeedback			
**VCR**	53.86 [40.12,67.86]	56.41 [33.28,66.79]	53.75 [38.19,65.83]
Sham biofeedback			
**VCR**	57.38 [40.92,67.94]	57.53 [36.94,68.42]	62.49 [42.85,69.21]

Legend to Supplementary Table S3

All values in median [interquartile range]. Interaction effects between group and time points of measurement were not significant (p>0.05) for VCR. Abbreviations: VCR, vasoconstrictory response
