# Supplementary material for: Heart rate variability biofeedback for critical illness polyneuropathy: a randomized sham‐controlled study
Source: Eur J Neurol. 2024 Oct 18;31(12):e16512. doi: 10.1111/ene.16512 (PMC11554868; doi:10.1111/ene.16512)
Supplement: Supplementary file 7 — Table S4: [file ENE-31-e16512-s003.pdf]

Supplementary Table S4 Sudomotor sympathetic skin response

	**Baseline**	**Post-intervention**	**Follow-up**
HRV biofeedback			
**SSR**	1.69 [0.48,3.61]	1.15 [0.37,3.09]	1.18 [0.32,4.65]
Sham biofeedback			
**SSR**	0.56 [0.07,2.33]	0.64 [0.06,5.41]	0.27 [0.13,0.70]

Legend to Supplementary Table S4

All values in median [interquartile range]. Interaction effects between group and time points of measurement were not significant ($p>0.05$) for SSR. Abbreviations: SSR, sympathetic skin response
